# Humor Styles as New Resources in a Primary Preventive Perspective: Reducing Resistance to Change for Negotiation

**DOI:** 10.3390/ijerph17072485

**Published:** 2020-04-05

**Authors:** Annamaria Di Fabio, Mirko Duradoni

**Affiliations:** 1 Department of Education, Languages, Intercultures, Literatures and Psychology (Psychology Section), University of Florence, 50135 Firenze, Italy; 2 Department of Information Engineering, University of Florence, 50139 Firenze, Italy; mirko.duradoni@unifi.it

**Keywords:** humor styles, resistance to change, negotiation, integrative negotiation, self-enhancing humor, affiliative humor, extraversion, emotionality

## Abstract

Reducing resistance to change is fundamental to dealing with the rapid and continuous changes of the 21st century labor market. Personality traits have been widely studied in relation to resistance to change. However, personality is not completely suitable for primary prevention intervention, since it does not change over time. Instead, humor styles appear to be a promising preventive resource to facilitate the negotiation process by enabling individuals to cope with the current work environment. Using a sample of 149 university students, this study analyzed the relationship between personality traits, such as extraversion and emotionality, humor styles, and resistance to change. The mediation analysis highlighted that both affiliative and self-enhancing humor styles could promote integrative negotiations within organizations in relation to change, due to their negative relationships with resistance to change. Thus, implementing dedicated interventions to increase the usage of affiliative and self-enhancing humor styles could help in lowering the failure risk in negotiation processes, supporting changes.

## 1. Introduction

Our century is characterized by rapid and incessant economic change. Knowing how to adaptively respond to change not only defines the longevity and success of an organization [1,2] but also ensures organizational well-being in line with the sustainable development goals defined by the UN [3]. In this sense, primary prevention interventions to build individuals’ strengths, such as the ability to adapt and promote change, as well as to reduce risks, are fundamental [4,5,6]. Even if change is crucial to adaptively cope with modern phenomena, such as globalization and unstable labor markets [7,8], change could be experienced by individuals as stressful and undesirable. Even though resistance to change is a natural part of the change process [9], the ability and willingness of individuals to adapt to organizational change differs [10]. Some studies suggest that loss of control due to change is one of the major causes of resistance [11]. Indeed, the feeling of losing control over life and work situations due to change (i.e., performing outside of a well-defined and familiar framework) can push individuals to oppose change. Lack of psychological resilience can hinder change processes too, since individuals with a low psychological resilience have worse coping strategies than their peers [12]. Change implies more work in the short term (i.e., learning and adjustments are required to adapt to new tasks) and people, especially those with a low psychological resilience, are reluctant to undergo the required adjustments [13]. Moreover, personality traits differences in resistance to change have been assessed by the scientific literature. For instance, if dogmatism emerged to predict individuals’ acceptance to change [14] in the past, more recent works have focused on emotional stability (i.e., emotionality, neuroticism) and extraversion traits [15,16]. These studies report that extraversion scores were negatively related to resistance to change scores, while emotionality (i.e., the tendency to experience anxiety in response to life’s stresses), appeared to be positively related with individuals’ reluctance to accept organizational changes. The existence of such relationships is not surprising since individuals with a high score on emotionality traits experience anxiety in response to life’s stresses [17]. Thus, changes within organizations could increase their levels of insecurity and produce further stress [16]. Extraversion, instead, is characterized by a high need for stimulation and extraverted individuals are more likely to welcome change than to resist it, i.e., they experience more positive emotions in relation to change [17,18]. Despite the evidence that links individuals’ dispositions, such as emotionality and extraversion, with workers’ resistance to change, personality is an intrinsic psychological feature that is unlikely to change over time. Traditionally, it has been considered as stable [19], not increasable through specific training. For this reason, the scientific literature regarding change dynamics within organizations, in line with the primary prevention approach, has recently considered other constructs and dynamics to facilitate interventions [20]. Traditionally, prevention is articulated in three levels: Primary prevention, secondary prevention, and tertiary prevention [21]. Primary prevention is focused on both avoiding the emergence of a problem before it begins and on promoting strengths. Secondary prevention regards early interventions when symptoms first emerge. Tertiary prevention aims to decrease symptoms and to support the functional recovery of the individual. The preventive perspective is more effective when the efforts to decrease risks are combined with the efforts to increase resources [5,6]. Primary prevention is particularly focused on building resources for individuals [4,5,6,22,23]. In this sense, in a constantly changing world, negotiation processes are crucial in organizations. Having new resources to reduce resistance to change could lead to a lower risk of failure for the negotiation processes [24,25].

Negotiation is an important area of research for organizational management [26] and can be defined as a decision-making form in which two or more parties interact with each other to resolve their opposing interests [27]. The following conditions are necessary to realize a negotiation process [28]: There are two or more parties (individuals, groups, organizations); there is a conflict between the needs and expectations of the parties; the parties choose to negotiate; there is the activation of a “give and take” process (the parties are willing to modify statements and initial requests to reach an agreement); and the parties prefer to negotiate to find an agreement instead of opposing. Change emerges as a pivotal condition for the negotiation process that seeks to reach an agreement among the different parties. In the literature, it is possible to distinguish between two perspectives [29]: Negotiation for win or distributive bargaining; and integrative negotiation or negotiation to grow. Negotiation for win or distributive bargaining regards bargaining situations that distribute, spread, or divide resources among the parties involved, with a perspective of constant or zero-sum power. Integrative negotiation, or negotiation to grow, is a decision-making process in which two or more parties interact to resolve or manage their opposite interests, with a perspective of variable sum power. The second form of negotiation, variable sum negotiation, is preferable to distributive because integrative builds long-term relationships (e.g., trust increased) and thus, facilitates working together in the future [30]. However, indications on how to conduct a negotiation that reduces resistance to change are scarce, at least regarding which type of communication is more effective and appropriate [31,32,33]. The Psychology of Harmony and Harmonization [34] asks instead for people’s relationality aspects which may be crucial in realizing harmony between individuals in different contexts, and the workplace is no exception.

Humor appears to be a resource which can be used to overcome resistance to change [35,36]. However, not all the humor styles enhance an openness towards change. Indeed, it has been reported by the scientific literature that a difference between potentially adaptive and beneficial functions of humor and the use of humor can be detrimental to well-being [15,37,38]. Aggressive humor (i.e., the expression of humor without regard for its potential impact on others) and self-defeating humor (i.e., excessively self-disparaging humor) appear to increase resistance to change. Differently, affiliative humor (i.e., the use of humor to facilitate relationships) and self-enhancing humor (i.e., the capability to maintain a humorous perspective even in the face of stressors) appear to benefit the change process [39].

Interestingly, affiliative humor is strongly correlated to extraversion [39,40], while self-enhancing humor is related to extraversion (positively) and emotionality (negatively) [39,41]. Therefore, humor styles are related both to personality traits (such as emotionality and extraversion) and resistance to change.

The article is organized as follows: First, the aim of the study is defined, and the hypotheses developed is based on the literature. Then, in the “Methods and Procedure” section information about the participants, their recruitment, the measures employed, and the data collection procedure are presented. In the Results section, both descriptive and inferential analyses are described. Finally, the discussion follows highlighting the strengths and limitations of the study.

### Aim of the Study and Hypotheses Development

The present study tests whether humor styles can mediate the relationship between personality traits (i.e., extraversion and emotionality) and individuals’ resistance to change. Indeed, evidence in the literature links humor styles with both personality traits and resistance to change. Nevertheless, none of the previous studies have considered the relationships of these three variables at the same time. First, we will test the assumption for mediation analysis by extending the literature results regarding the relation between emotionality and extraversion traits and resistance to change [15,16] by employing the HEXACO model of personality traits, which on the contrary has never been tested together with resistance to change. Starting from the Eysenck three-factor model of personality [42,43], and passing through the well-known and established Five-Factor model [44,45], the HEXACO model of personality can be conceived as the most evolved and updated conceptualization of personality [17,46].

Despite the lack of evidence, based on the Five-Factor model of personality, we can draw a link between emotionality and extraversion traits and resistance to change [15,16]. In particular, the emotionality trait should be positively associated with resistance to change (H2), while extraversion negatively (H5).

The relationship between the HEXACO model of personality traits and humor styles as defined by Martin et al. [39] has been only tested once [47]. Referring to this study, we hypothesized that emotionality could be negatively associated with self-enhancing humor (H1). The extraversion trait should instead be positively correlated with both affiliative (H3) and self-enhancing humor (H4).

As for the connection between humor and resistance to change, no other work tested it referring to the model of humor by Martin et al. Previous literature regarding other models stressed the role of “adaptive” humor in reducing resistance to change [35,39]. Thus, we transposed this evidence to the model of humor by Martin et al. in formulating our hypotheses, considering self-enhancing and affiliative humor styles as an “adaptive” form of humor. Consequently, we expected a positive correlation between resistance to change and both self-enhancing (H6) and affiliative (H7) humor styles, while no relationship is expected regarding “maladaptive” humor styles (H11).

Finally, given the expected relationships, in this study we will also construct mediation models using personality traits as predictors (i.e., emotionality and extraversion), humor styles as mediators (self-enhancing and affiliative humor), and individuals’ resistance to change as outcome variable (H9 and H10). The exploration of the mechanisms through which the HEXACO model of personality traits (causal variables) affects resistance to change (both directly and indirectly) has never been tested so far in the literature.

In the end, the following hypotheses were formulated:

**H1**: *Emotionality is negatively correlated with self-enhancing humor.*

**H2**: *Emotionality is positively correlated with individuals’ resistance to change.*

**H3**: *Extraversion is positively correlated with affiliative humor.*

**H4**: *Extraversion is positively correlated with self-enhancing humor.*

**H5**: *Extraversion is positively correlated with individuals’ resistance to change.*

**H6**: *Self-enhancing humor is negatively correlated with resistance to change.*

**H7**: *Affiliative humor is negatively correlated with resistance to change.*

**H8**: *Self-enhancing humor mediates the effect of emotionality on individuals’ resistance to change.*

**H9**: *Self-enhancing humor mediates the effect of extraversion on individuals’ resistance to change.*

**H10**: *Affiliative humor mediates the effect of extraversion on individuals’ resistance to change.*

**H11**: *Aggressive humor and self-defeating humor are not able to mediate the relationship between the personality traits considered (i.e., emotionality and extraversion) and resistance to change.*

## 2. Materials and Methods

### 2.1. Participants

Given the exploratory nature of the present work, the authors chose a nonprobability method based on the voluntary census to test the hypotheses. In these circumstances, studies based on voluntary participation can be extremely effective [48]. Relying on descriptive statistics and correlation matrices shown in previous similar studies, we determined the necessary sample size to conduct our analyses. Assuming the same relationship between the variables that we want to test in our mediation models and a significance level of 0.05, we carried out the Monte Carlo Power Analysis for Indirect Effects [49]. The analysis showed that a sample size of 110 individuals would be enough to ensure a statistical power of 0.80. Overall, 149 (109 females) participants were recruited for this study. The participants were the University of Florence’s students, with an average age of 24 (sd = 2.2), that soon will approach the world of work.

### 2.2. Measures

#### 2.2.1. HEXACO-60—Italian Version

HEXACO [46] is a short personality inventory composed of 60 items that are measured via a 5-point Likert scale, ranging from 1 = absolutely false to 5 = absolutely true. The HEXACO assesses six dimensions: Honesty-humility; emotionality (e.g., I would feel afraid if I had to travel in bad weather conditions); extraversion (e.g., I prefer jobs that involve active social interaction to those that involve working alone); agreeableness; conscientiousness; and openness to experience. The reliability coefficients for the HEXACO scale ranged between 0.73 and 0.80. The psychometric properties and the validity of the Italian version of HEXACO resulted in line with the original scale [50].

#### 2.2.2. Resistance to Change Scale—Italian Version

Derived from the original scale [15], the Italian version of the Resistance to Change Scale (RCS) is composed of 18 items measured via a 5-point Likert scale, ranging from 1 = absolutely false to 5 = absolutely true. Different from the original scale, the Italian version of the instrument is articulated in three dimensions instead of four, namely: Routine Seeking (e.g., I will take a routine day over a day full of unexpected events any time), Emotional Reaction to Imposed Change (e.g., If I were to be informed that there is going to be a significant change regarding the way things are done at work, I would probably feel stressed), and Cognitive Rigidity (e.g., I do not change my mind easily). As was true of the original scale, the Italian version of the Resistance to Change Scale is able to produce a total score, with higher scores signaling a lower readiness and willingness to change. The reliability coefficients for the original RCS dimensions ranged between 0.74 and 0.84 (Cronbach’s alpha = 0.87 for the RCS total score). The psychometric properties of the Italian version were assessed and showed acceptable results [51].

#### 2.2.3. Humor Styles Questionnaire—Italian Version

The Humor Style Questionnaire (HSQ) is composed of 32 items (eight items for each dimension), measured through a 7-point Likert scale, ranging from 1 = totally disagree to 7 = totally agree [39]. The HSQ assesses four dimensions related to individual differences in the use of humor. These are: Self-enhancing humor (i.e., humor is used to enhance the self), affiliative humor (i.e., humor fulfils the role of enhancing social relationships), aggressive humor (i.e., humor serves to strengthen the self at the expense of others), and self-defeating (i.e., humor is used to improve relationships at the expense of self). The reliability of the scale ranges from 0.77 to 0.81. The psychometric properties of the Italian adaptation of HSQ appear in line with the original scale [52].

### 2.3. Procedure

The questionnaires were administered to the students in group sessions by trained psychologists. The study assured respondents anonymity and confidentiality. The questionnaire included a statement (i.e., informed consent) regarding the personal data treatment, in accordance with the Italian privacy law (Law Decree DL-196/2003). The workers authorized and approved the use of anonymous/collective data for possible future scientific publications. Any participant could withdraw from the data collection session at any time. The questionnaires were administered in a counterbalanced order to control for order effects.

### 2.4. Data Analysis

We first verified the preconditions necessary for Pearson correlation and mediation analysis. For each Pearson correlation, we assessed the variables’ normality (asymmetry and kurtosis values), homoscedasticity, and linearity. We also excluded gender-related differences for mediators and dependent variables through independent sample Student t-tests. Subsequently, we carried out the regression procedures supported by Hayes [53] for the assessment of mediation using the PROCESS version 3.2 for SPSS [54]. We chose this statistical method since it allows to understand the mechanisms through which the causal variable affects the outcome (both directly and indirectly). We tested several simple mediation models (i.e., theoretical model 4) to assess the causal effects of an independent variable (X) on an outcome variable (Y) through an intervening mediator variable (M). According to Hayes [53], in a simple mediation model there are two possible pathways in which X could affect Y. The first path directly connects X to Y, while the second bridges X and Y indirectly through M. Then, we proceeded to estimate the effect sizes of the mediator through the index of mediation (i.e., completely standardized indirect effect) and k^2^ coefficient.

## 3. Results

### 3.1. Descriptive Statistics

At first, we described our sample using the participants’ average and standard deviation for personality traits, humor styles, and resistance to change. Table 1 reports the descriptive statistics for our University students’ sample. The data refer to all dimensions involved in our data collection.

### 3.2. Mediation Analysis

Correlations between personality traits, Humor Style dimensions, and Resistance to Change scores were explored. As a first step, we decided to not only test our first set of hypotheses (H2 and H5) but also to give the full picture of the relationship between the HEXACO model of personality and resistance to change by means of Pearson correlation. In general, both Extraversion and Emotionality appeared related to Resistance to change. The results are shown in Table 2.

As expected, Emotionality and Extraversion obtained the highest correlations among personality traits and thus, were suitable as possible predictors for our mediation modeling procedures. Subsequently, we tested the relationship between Emotionality and Extraversion with Humor Style dimensions to test H1, H3, and H4 hypotheses. Emotionality showed a negative linear relationship with Self-Enhancing Humor (r = −0.29; *p* = 0.001) and Aggressive Humor (r = −0.36; *p* = 0.001). Nevertheless, Aggressive Humor did not show a significant linear relationship with Resistance to Change scores. Thus, Aggressive Humor style was not suitable as a possible mediator because preconditions for the mediation analysis were not met and thus confirming H11. Differently, Self-Enhancing Humor correlated with Resistance to Change scores (r = −0.42; *p* = −0.001). Extraversion presented a positive relationship with Affiliative Humor (r = 0.45; *p* = 0.001) and Self-Enhancing Humor (r = 0.28; *p* = 0.001). Affiliative Humor appeared correlated with Resistance to Change scores (r = 0.45; *p* = 0.001). Overall, both aggressive humor and self-defeating humor appear not to be suitable for mediation analysis (H6).

**Figure 1:**
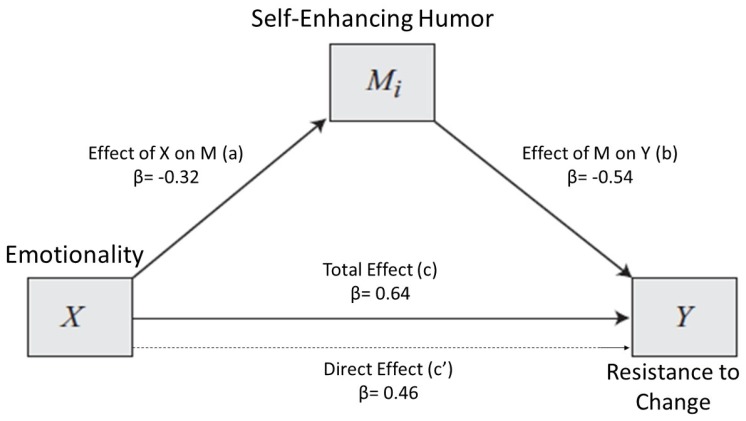
Model 1. Relationship between Emotionality and Resistance to Change, with Self-Enhancing Humor as a mediator. k^2^ mediator effect size = 0.10.

On this basis, we decided to perform three mediation analyses in order to test our last three hypotheses H8, H9, and H10. The first model considered Emotionality as an independent variable and Self-Enhancing Humor as a mediator to predict Resistance to Change (model 1). The second and third model had the same independent variable (i.e., Extraversion) and criterion variable (i.e., Resistance to Change) but different mediators; Affiliative Humor for model 2 and Self-Enhancing Humor for model 3. Figure 1 represents the relationship between Emotionality and Resistance to Change with Self-Enhancing Humor as a mediator. Generally, Self-Enhancing Humor appeared able to mediate the effects of Emotionality on Resistance to Change. The statistics related to each mediation path are presented in Table 3.

Consistent with our hypothesis, Emotionality showed a direct positive influence on Resistance to Change (path c’). In other words, high-Emotionality individuals reported more difficulties in accepting change. Moreover, Emotionality appeared to influence Resistance to Change scores indirectly through the Self-Enhancing Humor style. Participants with a lower level of Emotionality were more likely to engage in Self-Enhancing Humor (path a) and this type of humor appears to be associated with a decrease in Resistance to Change (path b).

In Table 4, model effects indices are summarized.

As we can gather from Table 4, 10% of the variance in Resistance to Change score is explained by the indirect effect.

**Figure 2:**
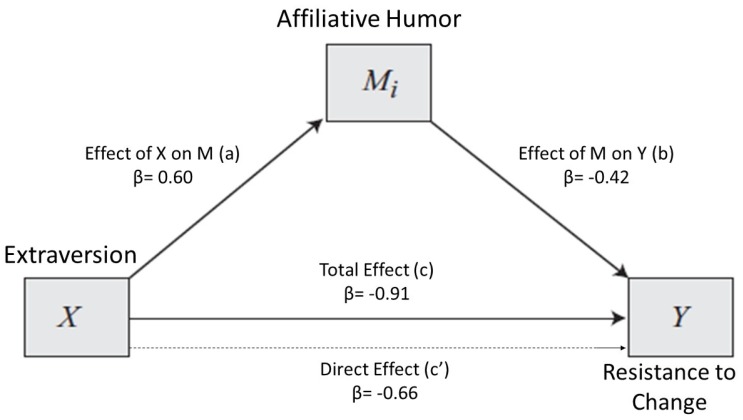
Model 2. Relationship between Extraversion and Resistance to Change, with Affiliative Humor as a mediator. k^2^ mediator effect size = 0.13.

With the second model, we tested if the Extraversion effect on Resistance to Change could be mediated by Affiliative Humor. In this case, Affiliative humor appeared able to mediate the Extraversion effect on Resistance to change. In Figure 2, the model 2 graphical representation is shown. In Table 5, the model related statistics are presented.

Consistent with our predictions, Extraversion demonstrated a significant direct negative effect on Resistance to Change (path c’). Indeed, highly extraverted individuals were more willing to accept change. Extraversion affected the criterion variable indirectly through Affiliative Humor. Participants who scored high on the Extraversion dimension were also more likely to use humor to enhance social relationships (path a). At the same time, Affiliative Humor appeared able to decrease the resistance connected to change (path b).

In Table 6, the model effects indices for model 2 are summarized.

As we can gather from Table 6, around 13% of the variance in Resistance to Change is explained by using Affiliative Humor as a mediator.

**Figure 3:**
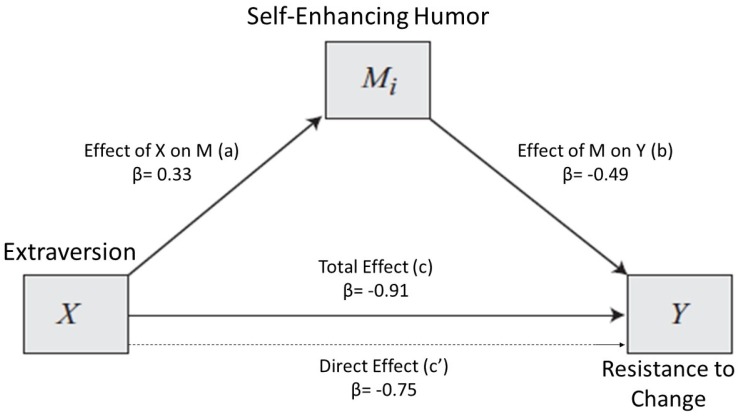
Model 3. Relationship between Extraversion and Resistance to Change, with Self-Enhancing Humor as a mediator. k^2^ mediator effect size = 0.09.

For the third model, we proceeded as before (i.e., maintaining both the independent and the criterion variable in the same way), testing for another mediator (i.e., Self-Enhancing Humor). Again, Self-Enhancing Humor resulted in being able to mediate the Extraversion effect on Resistance to change. We presented the model 3 graphical representation in Figure 3 and its related statistics in Table 7.

Not surprisingly, the direct relationship between Extraversion and Resistance to Change remained consistent with what the previous model has shown (path c’). As before, highly extraverted participants had lower Resistance to Change scores (i.e., were more prone to change). Extraversion also appeared to act on Resistance to Change indirectly through Self-Enhancing Humor. Highly extraverted individuals reported a more frequent use of Self-Enhancing Humor style (path a) than their peers. At the same time, the use of Self-Enhancing Humor seems to decrease individuals’ Resistance to Change (path b).

In Table 8, the model effects indices for model 3 are shown.

As shown in Table 8, 9% of the Resistance to Change scores appear to be explained by a Self-Enhancing Humor indirect effect.

Finally, since Affiliative Humor and Self-Enhancing Humor styles are related (r = 0.48; *p* = 0.001), we explored the possibility of a double mediator model (i.e., theoretical model 6). However, this type of mediator model result was inadequate to explain our data. In particular, the d path (i.e., the indirect effect that passes through both mediators) showed a completely standardized indirect effect lower than 2%, suggesting that there is no support for a double mediation structure. Thus, both Self-Enhancing and Affiliative Humor styles seem to be independent mediators of the relationship between Extraversion and Resistance to Change.

## 4. Discussion

In general, the current study extends previous research by examining mediation models that explain resistance to change and assesses the role of humor styles as a mediator of extraversion and emotionality among young adults. Overall, self-enhancing humor appeared able to mediate both the effects of the Extraversion and Emotionality traits on Resistance to Change. In addition, the Affiliative humor appeared able to mediate the Extraversion effect on Resistance to Change, thus underlying the humor styles’ potential in lowering resistance to change.

First, we analyzed and confirmed the literature results concerning the relationships between emotionality, self-enhancing humor, and individuals’ resistance to change [16,41] (H1–H2). Secondly, the relationship between extraversion, self-enhancing, and affiliative humor and resistance to change was analyzed and confirmed [15,16,39] (H3–H5). Hypotheses 6 and 7 (H6–H7) were also supported, confirming the negative relationship between self-enhancing and affiliative humor and resistance to change [35,39]. Finally, the three mediation models accounted by hypotheses 8, 9, and 10 (H8–H9–H10), resulted as adequate. Self-enhancing humor appears to mediate both the effects of emotionality (H8) and extraversion (H9) on participants’ resistance to change, while affiliative humor seemed to mediate the effect of extraversion on individuals’ resistance to change (H10). Following the k^2^ interpretation [55], we have a medium mediator effect size for all the models. The summary of the confirmed hypotheses of the whole study is presented in Table 9.

Emotionality traits appear to be detrimental for both self-enhancing humor and the change process. This is not surprising since high emotionality individuals are connotated by a lack of resilience and have poor coping strategies (e.g., self-enhancing humor style) [56]. In other words, high emotionality individuals lack the resources, such as a self-enhancing humor style, which would be useful to break down resistance to change. Differently, extraverted individuals appeared particularly able to use both affiliative and self-enhancing humor styles. As before, this result is in line with the scientific literature. Indeed, extraverted people engage in social interaction with a positive affective style and tend to build and maintain strong networks of social support, which allows them to face and adapt to stressful situations (e.g., changes, negotiations) [56,57]. Comparing models 2 and 3, which share the same personality trait as an independent variable, it seems that affiliative humor can be the best adaptive strategy to reduce resistance to change for extraverted individuals (i.e., model 2 has the highest mediating effect). Nevertheless, the self-enhancing humor, as a useful resource for personal coping purposes, still showed adaptive results in terms of lowering resistance to change in both model 1 and 3, exhibiting a similar mediation effect size across the two personality traits considered (i.e., extraversion and emotionality).

Unlike personality traits, self-enhancing and affiliative humor styles are trainable and could more easily be used within a primary prevention perspective [4,5,6]. In other words, it seems possible to decrease workers’ resistance to change and, thus, to facilitate the negotiation process by means of specific trainings based on increasing the workers’ ability to engage in affiliative and self-enhancing humor styles. Our results could also contribute to define some relational aspects able to favor positive relationships with others and organizational contexts and thus foster processes of harmonization [34] in a constantly changing world.

Nevertheless, our work still is exploratory and correlational (i.e., causal relationships are not involved). Thus, future research should test if causal relationships exist between personality traits (i.e., extraversion, emotionality), humor styles (self-enhancing, affiliative), and resistance to change. Moreover, several further limitations of this study need to be addressed. The results of the present study are not generalizable to Italian workers, since the participants were the University of Florence’s students, albeit approaching the word of work. Moreover, the participants were almost women and gender surely influences emotionality [58]. Future research should, therefore, expand the investigation regarding the mediation effect of humor styles on resistance to change, involving workers’ samples too, and from different geographical areas in Italy, as well as from foreign countries to address the cross-cultural invariance of our results. Future research should also consider other possible relationships between humor styles and constructs that the scientific literature has suggested as able to decrease resistance to change. For instance, emotional intelligence [59] could potentially sustain the impact of affiliative and self-enhancing humor styles on resistance to change, providing workers with better coping strategies to face change, as well as to lead successful integrative negotiations. Moreover, the leadership style should be considered as an instrument with which to support workers’ humor style and their attitude towards change [60].

## 5. Conclusions

Despite its limitations, this study suggests that affiliative and self-enhancing humor styles could promote integrative negotiations within organizations, due to their negative relationships with resistance to change. Moreover, the emotions experienced during negotiation are a key factor for workers’ satisfaction [61]. Thus, using an appropriate humor style during the negotiation processes could positively affect workers’ mindset and attitudes towards future negotiations.

Overall, humor and humor styles can be conceived as a promising primary prevention resource for work and organizational contexts. More generally, humor and humor styles could be considered as a useful new preventive resource to promote employee health and safety, as well as organizational effectiveness and sustainability (i.e., healthy organization) [62,63,64,65,66,67,68,69,70,71,72,73,74,75,76].

## Figures and Tables

**Table 1:** Descriptive statistics for personality traits, humor style questionnaire’s dimensions, and resistance to change. The average and standard deviation is presented for each variable.

Scale	Variable	Average (sd)
HEXACO	Honesty-humility	33.89 (5.54)
	Emotionality	33.41 (6.56)
	Extraversion	33.37 (6.09)
	Agreeableness	30.06 (5.58)
	Conscientiousness	35.59 (5.46)
	Openness to experience	32.65 (5.64)
Humor Style	Affiliative Humor	41.33 (8.07)
	Self-Enhancing Humor	35.31 (7.29)
	Aggressive Humor	29.02 (8.35)
	Self-Defeating Humor	28.65 (7.65)
Resistance to Change	Total Score	50.98 (11.65)

**Table 2:** Pearson correlation between personality traits and Resistance to Change score.

Scale	Variable	Resistance to Change
HEXACO	Honesty-humility	0.14
	Emotionality	0.36 **
	Extraversion	−0.48 **
	Agreeableness	0.19 *
	Conscientiousness	−0.01
	Openness to experience	−0.22 **

*: *p* ≤ 0 05; **: *p* ≤ 0 01.

**Table 3:** Model 1. Mediation analysis statistics. In the upper part of the table, the indexes related to each regression analysis needed for testing the mediational model are summarized. In the lower part, the coefficients for each path of the mediation model are provided.

	F	df	*p*	R^2^
X predicts M	13.05	1147	0.001	0.08
X and M predict Y	22.74	2146	0.001	0.24
X predicts Y	21.97	1147	0.001	0.13
	Student t	df	*p*	β
Path a	−3.61	147	0.001	−0.32
Path b	−4.54	147	0.001	−0.54
Path c’	3.48	147	0.001	0.46
Path c	4.68	147	0.001	0.64

Note: df: degrees of freedom.

**Table 4:** Model 1 effect indices. In the table, the coefficients for all the effects of the mediation model number 1 are presented.

Total Effect	Direct Effect	Indirect Effect	Partial Standardized Indirect Effect	Total Standardized Indirect Effect	k^2^
0.64	0.47	0.17	0.015	0.09	0.10

**Table 5:** Model 2. Mediation analysis statistics. In the upper part of the table, the indexes related to each regression analysis needed for testing the mediational model are summarized. In the lower part, the coefficients for each path of the mediation model are provided.

	F	df	*p*	R^2^
X predicts M	38.32	1147	0.001	0.20
X and M predict Y	30.61	2146	0.001	0.30
X predicts Y	43.36	1147	0.001	0.23
	Student t	df	*p.*	β
Path a	6.19	147	0.001	0.60
Path b	−3.74	147	0.001	−0.42
Path c’	−4.41	147	0.001	−0.66
Path c	−6.58	147	0.001	−0.91

**Table 6:** Model 2 effect indices. In the table, the coefficients for all the effects of the mediation model number 2 are presented.

Total Effect	Direct Effect	Indirect Effect	Partial Standardized Indirect Effect	Total Standardized Indirect Effect	k^2^
−0.91	−0.66	−0.25	0.02	−0.13	0.13

**Table 7:** Model 3. Mediation analysis statistics. In the upper part of the table, the indexes related to each regression analysis needed for testing the mediational model are summarized. In the lower part, the coefficients for each path of the mediation model are provided.

	F	df	*p*	R^2^
X predicts M	12.41	1, 147	0.001	0.08
X and M predict Y	33.61	2, 146	0.001	0.31
X predicts Y	43.36	1, 147	0.001	0.23
	Student t	df	*p*	β
Path a	7.50	147	0.001	0.33
Path b	−4.32	147	0.001	−0.49
Path c’	−5.48	147	0.001	−0.75
Path c	−6.58	147	0.001	−0.91

**Table 8:** Model 3 effect indices. In the table, the coefficients for all the effects of the mediation model number 3 are presented.

Total Effect	Direct Effect	Indirect Effect	Partial Standardized Indirect Effect	Total Standardized Indirect Effect	k^2^
−0.91	−0.75	−0.16	−0.014	−0.09	0.09

**Table 9:** Summary of the confirmed hypotheses.

Hypotheses	Confirmed?
H1: Emotionality is negatively correlated with self-enhancing humor.	Yes
H2: Emotionality is positively correlated with individuals’ resistance to change.	Yes
H3: Extraversion is positively correlated with affiliative humor.	Yes
H4: Extraversion is positively correlated with self-enhancing humor.	Yes
H5: Extraversion is positively correlated with individuals’ resistance to change.	Yes
H6: Self-enhancing humor is negatively correlated with resistance to change.	Yes
H7: Affiliative humor is negatively correlated with resistance to change.	Yes
H8: Self-enhancing humor mediates the effect of emotionality on individuals’ resistance to change.	Yes
H9: Self-enhancing humor mediates the effect of extraversion on individuals’ resistance to change.	Yes
H10: Affiliative humor mediates the effect of extraversion on individuals’ resistance to change.	Yes
H11: Aggressive humor and self-defeating humor are not able to mediate the relationship between the personality traits considered (i.e., emotionality and extraversion) and resistance to change.	Yes
